# N-WASP Is Essential for the Negative Regulation of B Cell Receptor Signaling

**DOI:** 10.1371/journal.pbio.1001704

**Published:** 2013-11-05

**Authors:** Chaohong Liu, Xiaoming Bai, Junfeng Wu, Shruti Sharma, Arpita Upadhyaya, Carin I. M. Dahlberg, Lisa S. Westerberg, Scott B. Snapper, Xiaodong Zhao, Wenxia Song

**Affiliations:** 1 Department of Cell Biology & Molecular Genetics, University of Maryland, College Park, Maryland, United States of America; 2 Division of Immunology, Children's Hospital of Chongqing Medical University, Chongqing, China; 3 Division of Infectious Diseases and Immunology, Department of Medicine, University of Massachusetts Medical School, Worcester, Massachusetts, United States of America; 4 Department of Physics, University of Maryland, College Park, Maryland, United States of America; 5 Translational Immunology Unit, Department of Medicine, Karolinska Institute, Stockholm, Sweden; 6 Department of Medicine, Harvard Medical School, Boston, Massachusetts, United States of America; Scripps Research Institute, United States of America

## Abstract

Negative regulation of receptor signaling is essential for controlling cell activation and differentiation. In B-lymphocytes, the down-regulation of B-cell antigen receptor (BCR) signaling is critical for suppressing the activation of self-reactive B cells; however, the mechanism underlying the negative regulation of signaling remains elusive. Using genetically manipulated mouse models and total internal reflection fluorescence microscopy, we demonstrate that neuronal Wiskott–Aldrich syndrome protein (N-WASP), which is coexpressed with WASP in all immune cells, is a critical negative regulator of B-cell signaling. B-cell–specific N-WASP gene deletion causes enhanced and prolonged BCR signaling and elevated levels of autoantibodies in the mouse serum. The increased signaling in N-WASP knockout B cells is concurrent with increased accumulation of F-actin at the B-cell surface, enhanced B-cell spreading on the antigen-presenting membrane, delayed B-cell contraction, inhibition in the merger of signaling active BCR microclusters into signaling inactive central clusters, and a blockage of BCR internalization. Upon BCR activation, WASP is activated first, followed by N-WASP in mouse and human primary B cells. The activation of N-WASP is suppressed by Bruton's tyrosine kinase-induced WASP activation, and is restored by the activation of SH2 domain-containing inositol 5-phosphatase that inhibits WASP activation. Our results reveal a new mechanism for the negative regulation of BCR signaling and broadly suggest an actin-mediated mechanism for signaling down-regulation.

## Abstract 2

A cell biology study using conditional gene knockout mouse models reveals a novel mechanism by which the actin cytoskeleton negatively regulates the signal transduction of the B cell antigen receptor.

## Author Summary

Mechanisms to shut down B-cell activation are necessary to ensure termination of an immune response when an infection has been cleared. When this negative regulation goes wrong, it can also lead to autoimmunity. To understand how this inhibitory process is regulated, here we utilized knockout mice containing B cells that are deficient for proteins potentially involved in their negative regulation. We focus on Wiskott–Aldrich syndrome protein (WASP), a key cytoskeletal regulator of hematopoietic cells, and neural WASP (N-WASP), which shares 50% homology with WASP and is ubiquitously expressed. Our study shows that mouse B cells that lack N-WASP protein are activated to a greater level and for longer periods than B cells that express this protein. Furthermore, in mice where B cells do not make N-WASP, the numbers of self-reactive B cells are elevated. We went on to identify molecules that promote or inhibit N-WASP activation and to examine the cellular mechanisms by which N-WASP inhibits B-cell activation. Based on these findings we propose that N-WASP is a critical inhibitor of B-cell activation and serves to suppress self-reactive B cells.

## Introduction

B lymphocytes are a key component of the immune system and responsible for generating antibody responses against foreign invaders. B-cell–mediated antibody responses are activated by signals generated from B-cell antigen receptor (BCR) and from T helper cells through antigen presentation. Antigen binding induces self-aggregation of BCRs into microclusters and BCR association with lipid rafts, which lead to the recruitment of signaling molecules to BCRs. First the tyrosine kinases Lyn and Syk are recruited followed by phospholipase Cγ2 (PLCγ2), phosphatidyinositol-3-kinase, Bruton's tyrosine kinase (Btk), and the guanine nucleotide exchange factor Vav for the GTPases Rac and Cdc42, which activate signaling cascades [1],[2]. BCR microclusters grow over time and subsequently merge into each other, resulting in the formation of a BCR central cluster at one pole of the cell [3]–[5]. After initial signaling activation, inhibitory signaling molecules, including the tyrosine and phosphatidylinositol phosphatases SHP, SHIP, and PTEN, are activated, down-regulating signaling [6]–[9]. Defects in the negative regulation of BCR signaling are associated with losses of B-cell self-tolerance and increases in the susceptibility to autoimmune diseases [10],[11]. However, the molecular details of the negative regulation of BCR signaling have not been well defined.

The self-clustering of surface BCRs into microclusters is an essential event for triggering signaling activation and a target for regulation. While surface BCRs have been shown to exist as tight but inhibitory oligomers at the nanoscale before activation [12],[13], antigen-induced coalescence and transformation of the nano-clusters into microclusters is required for BCR activation. Spontaneous formation of BCR microclusters induced by actin depolymerization leads to signaling activation in the absence of antigen [14]–[16]. BCRs with high affinity to an antigen cluster induce signaling with faster kinetics and to higher levels than those with low affinity to the antigen [17],[18]. Conversely, the co-engagement of the BCR with the inhibitory coreceptor FcγRIIB by antigen–antibody complexes inhibits both BCR clustering and signaling [19],[20]. We have recently shown that while the formation of BCR microclusters induces signaling, the coalescence of BCR microclusters into the BCR central cluster is associated with signaling attenuation at the B-cell surface. Both the attenuation of BCR signaling and the coalescence of BCR microclusters into the central cluster are inhibited in B cells where the gene of SH2 domain-containing inositol 5-phosphatase (SHIP-1) is specifically deleted [21]. These results suggest that the formation of the BCR central cluster is a down-regulatory mechanism for BCR signaling.

BCR self-clustering at the B-cell surface depends on actin reorganization. Actin can regulate BCR clustering by controlling the lateral mobility of surface receptors and B-cell morphology [5],[15],[17],[22]. Perturbing the cortical actin increases the lateral mobility of surface BCRs and facilitates BCR self-clustering and BCR signaling [15], while stabilizing the actin network does the opposite [16]. In response to membrane-associated antigen, B cells undergo actin-dependent spreading and contraction. B-cell spreading expands the area of contact between the B-cell surface and the antigen-tethered membrane, thereby increasing the number of antigen-engaged BCRs [17]. The merger of BCR microclusters into the central cluster appears to depend on B-cell contraction following spreading since the BCR central cluster fails to form when cell contraction is inhibited [21]. Conversely, BCR clustering and B-cell spreading are regulated by BCR signaling. B cells with genetic deletion of signaling molecules, CD19, PLCγ2, Btk, Vav, or Rac, exhibit impaired BCR clustering and B-cell spreading [23]–[25]. We have demonstrated that the stimulatory kinase Btk is essential for the activation of the actin regulator Wiskott–Aldrich syndrome protein (WASP), B-cell spreading, and BCR clustering. In contrast, the inhibitory phosphatase SHIP-1 inhibits WASP activation by suppressing Btk activation, which promotes B-cell contraction and the coalescence of BCR microclusters into the central cluster [21]. Furthermore, actin reorganization is essential for BCR internalization [26], which is not only the initiation step of antigen processing but also a mechanism for down-regulation of receptor signaling. Therefore, actin can provide both positive and negative feedback to BCR signaling.

WASP is an actin-nucleation–promoting factor that is specifically expressed in hematopoietic cells [27],[28]. WASP mutations that ablate its expression cause a severe and complicated X-linked immune disorder, WAS [29]–[31], which demonstrates the critical role of actin in immune regulation. WAS patients suffer from recurrent bacterial infections, which are associated with defective T-independent antibody responses against polysaccharide and impaired maturation of T-dependent antibody responses [30],[32]–[34]. In addition, a large portion of WAS patients develop autoimmune diseases, which are associated with a higher risk of leukemia and lymphoma [35],[36]. In the WASP knockout (KO) mouse model, there is no significant defect in T- and B-cell development, except for a reduction in marginal zone B cells [37]–[39]. The in vitro activation of T cells but not B cells from WASP KO mice is decreased [40]. WASP-deficient B cells from both mice and WAS patients exhibit defective migration [34],[37],[38]. We have found that there were significant reductions in cell spreading, BCR clustering, surface tyrosine phosphorylation [21], and BCR internalization [41] in WASP KO B cells as compared to those of wild type (wt) B cells. Recent studies have clearly demonstrated a critical and B-cell–intrinsic role for WASP in controlling B-cell self-tolerance [39],[41]. However, the molecular mechanism underlying the loss of self-tolerance in WASP-deficient B cells has not yet been elucidated.

In addition to hematopoietic-specific WASP, B cells, like all immune cells, also express the ubiquitous N-WASP. These two proteins share ∼50% sequence homology [42] and are reported to have the same cellular function, activating actin polymerization and branching by binding to Arp2/3 complexes [43],[44]. They are capable of linking cell signaling to actin dynamics via their GTPase binding (GBD), proline rich (PRD), and pleckstrin homology (PH) domains. The binding of GTP–Cdc42 and phosphatidylinositol-4,5-biphosphate (PI(4,5)P_2_) releases the proteins from an autoinhibitory conformation [43],[45],[46]. Signaling-induced phosphorylation of WASP and N-WASP at tyrosines in the GBD domain and serines in the VCA (verprolin homology, cofilin homology, and acidic) domain is required for their optimal activity [47]–[49]. In B cells, Btk is responsible for activating WASP by activating Vav, a guanine nucleotide exchange factor for Cdc42, stimulating the production of PI(4,5)P_2_, and inducing the phosphorylation of WASP [50]. While WASP and N-WASP have been well studied individually, how these two proteins function when coexpressed is largely unknown. Recent studies have demonstrated that both WASP and N-WASP are critical for the development and function of B cells [38]. However, why both WASP and N-WASP are required and how N-WASP and WASP functionally coordinate with each other during BCR activation is not well understood.

In this study, we examined the cellular function of N-WASP and its functional relationship with WASP during BCR activation using WASP KO, B-cell–specific N-WASP KO, and double KO mice, as well as primary human B cells from WAS patients. We found that while both WASP and N-WASP are required for optimal signaling activation and internalization of the BCR, the two proteins have distinct functions. N-WASP is critical for the down-regulation of BCR signaling. N-WASP promotes signaling attenuation by facilitating B-cell contraction and coalescence of BCR microclusters into a central cluster and by mediating BCR internalization. Surprisingly, WASP and N-WASP functionally suppress each other and are inversely regulated by stimulatory and inhibitory signals. These results reveal a new function for N-WASP in the negative regulation of receptor signaling and demonstrate a unique functional relationship between N-WASP and WASP during BCR activation.

## Results

### N-WASP Is Activated Following WASP Activation in Response to BCR Stimulation

**Figure 1:**
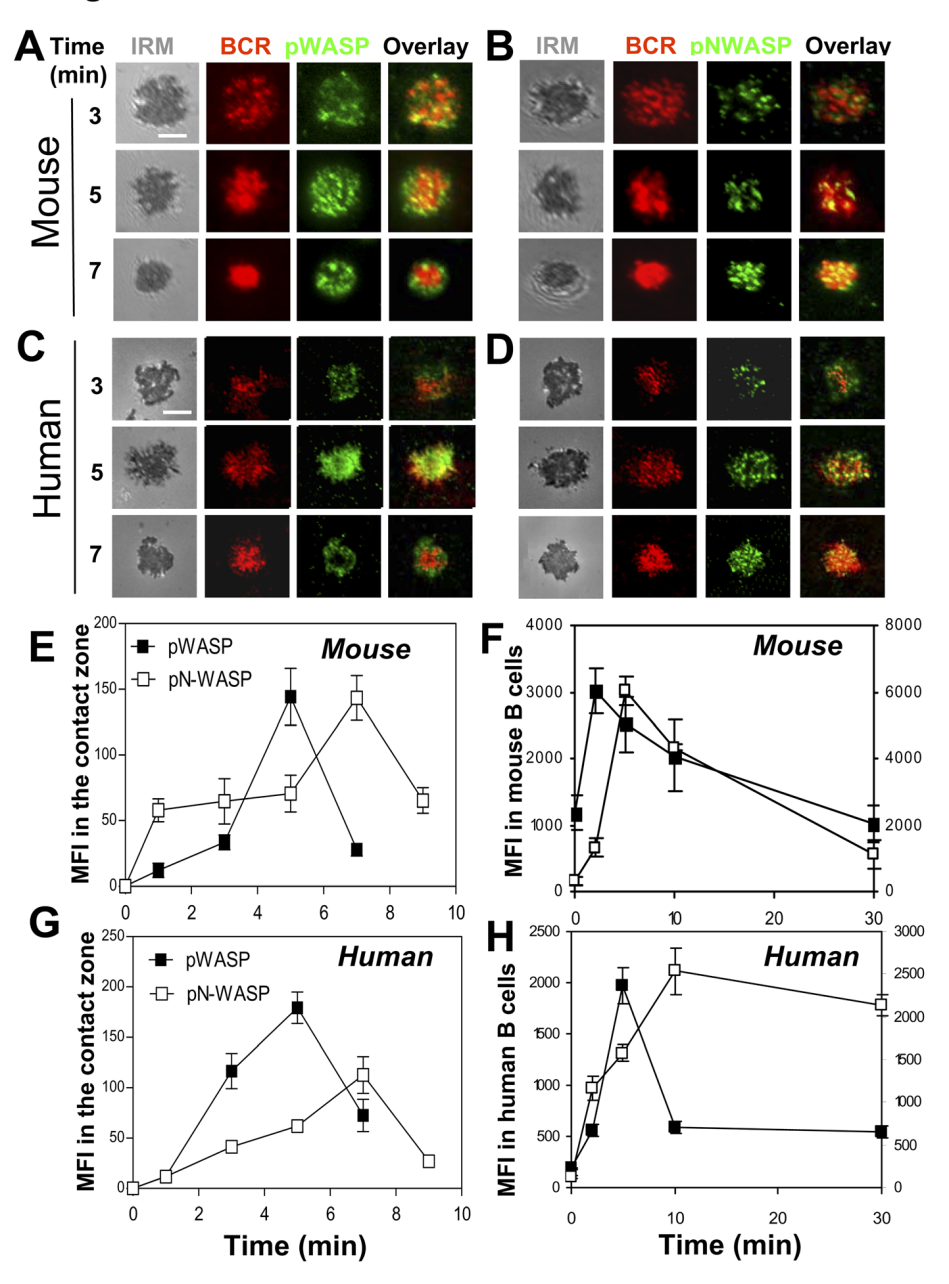
N-WASP is activated following WASP activation upon antigen stimulation. (A–D) TIRFM and IRM analysis of pWASP and pN-WASP in the B-cell contact zone of mouse splenic B cells and human PBMC B cells that were incubated with membrane-tethered AF546–mB-Fab′–anti-mouse or human IgG+M at 37°C for indicated times. (E and G) The MFI of pWASP or pN-WASP in the B-cell contact zone was quantified using TIRFM images and Andor iQ software. (F and H) The MFI of pWASP or pN-WASP in mouse splenic and human PBMC B cells incubated with soluble Fab′–anti-IgG+M plus streptavidin at 37°C for indicated times were analyzed by flow cytometry. Shown are representative images and the average MFI (±SD) from three independent experiments. Bar, 2.5 µm.

To understand the function of N-WASP in BCR activation, we examined the activation status of N-WASP in relation to WASP activation in human peripheral blood (PBMC) and mouse splenic B cells in response to BCR cross-linking. We labeled and cross-linked surface BCRs using Alexa Fluor 546–labeled, monobiotinylated Fab′ fragment of anti-mouse or human IgG+M antibody (AF546–mB-Fab′–anti-Ig) that was tethered to planar lipid bilayers by streptavidin to mimic membrane-associated antigen or mB-Fab′–anti-Ig plus soluble streptavidin to mimic soluble antigen. We chose this activation system because it activates the BCR on mouse and human B cells in a similar way and because it has similar capability of inducing BCR activation as bona fide Ag, such as hen egg lysozyme (HEL) tethered to lipid bilayers that activates B cells from MD4 transgenic mice (Figure S1). Active WASP and N-WASP were detected using antibodies specific for their phosphorylated forms that did not show cross-reactivity between phosphorylated WASP and N-WASP (Figure S2). Human and mouse B cells were activated with membrane-tethered or soluble mB-Fab′–anti-Ig plus streptavidin before staining for phosphorylated WASP (pWASP) and N-WASP (pN-WASP). Using total internal reflection fluorescence microscopy (TIRFM), we analyzed the distribution and the relative levels of pWASP and pN-WASP at the surface of B cells in contact with the mB-Fab′–anti-Ig–tethered lipid bilayer (B-cell contact zone). We found that both pWASP and pN-WASP were detected in the contact zone of mouse (Figure 1A,B) and human B cells (Figure 1C,D), and they have a punctate appearance. As Ag–BCR complexes coalesced and merged into a central cluster accompanied by B-cell contraction, most of the pWASP staining (Figure 1A,C), but not pN-WASP (Figure 1B,D), moved away from the center to the edge of antigen–BCR central clusters. The mean fluorescence intensity (MFI) of pN-WASP in the B-cell contact zone increased over time similar to that of pWASP. However, the pN-WASP MFI reached its maximal level 2 min later than pWASP, concurrent with the pWASP levels returning to the basal levels (Figure 1E,G). Flow cytometry analysis was used to determine the overall levels of pWASP and pN-WASP in response to mB-Fab′-anti-Ig plus soluble streptavidin. We found similar patterns of WASP and N-WASP activation, where the MFI of pN-WASP peaked 3–5 min later than that of pWASP until the pWASP levels decreased (Figure 1F,H). These results indicate that N-WASP is transiently activated at locations where BCRs interact with antigen similar to WASP, but its activation does not peak until the level of WASP activation decreases.

### Both WASP and N-WASP Are Critical for Antigen-Induced BCR Clustering and B-Cell Morphological Changes but the Two Play Distinct Roles

**Figure 2:**
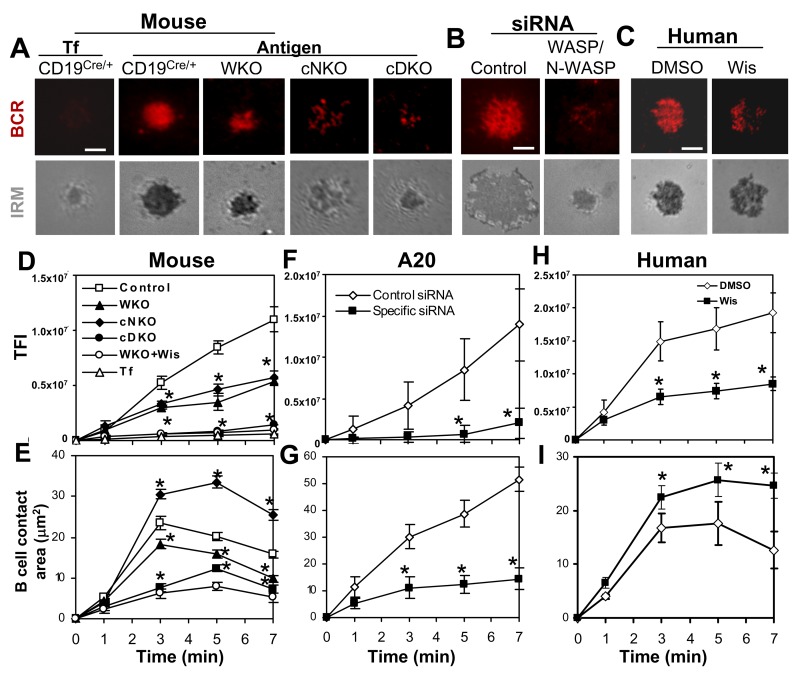
Antigen-induced BCR clustering and B-cell spreading depend on both WASP and N-WASP. (A–C) TIRFM and IRM analysis of mouse splenic B cells that were incubated with membrane-tethered transferrin (Tf) or Fab′–anti-Ig (A), A20 B cells that were transfected with control or WASP/N-WASP siRNA (B), and human B cells that were pretreated with or without wiskostatin (Wis) and stimulated with membrane-tethered Fab′–anti-Ig (C). Shown are representative images from 7 min. Bar, 2.5 µm. (D–I) The average values (±SD) of the TFI of Fab′–anti-Ig in the B-cell contact zone (D, F, and H) and of the B-cell contact area (E, G, and I) were determined using TIRFM and IRM images from >300 individual cells of 18 mice for each data point including littermate controls (D–E) or of three individual experiments (F–G and H–I). *p<0.01, compared to B cells from littermate control mice, transfected with control siRNA or treated with DMSO.

Previous studies have shown that WASP is dispensable for antigen-induced BCR clustering and B-cell morphological changes [21],[41], implying a compensatory role for N-WASP in WASP KO B cells. To investigate this hypothesis, we utilized WASP KO mice (WKO), B-cell–specific N-WASP KO mice (cNKO), and double KO mice where N-WASP is selectively deleted in B cells (cDKO), established previously by Westerberg et al. [38]. Using reverse transcription PCR and Western blot, we were unable to detect any mRNA and protein of N-WASP in B cells sorted from the splenocytes of cNKO and cDKO mice (Figure S3), confirming the effective deletion of *loxP* flanked *n-wasp* gene by CD19^Cre^. We examined the effect of WASP and/or N-WASP KO on BCR clustering and B-cell morphology in response to membrane-tethered Fab′–anti-Ig. Surface BCR clustering was analyzed by TIRFM. As we have shown previously [16],[21], upon being bound by the BCR, AF546–mB-Fab′–anti-Ig clustered, coalesced, and formed a polarized central cluster at 7 min (Figure 2A), and the total fluorescence intensity (TFI) of AF546–mB-Fab′–anti-Ig in the contact zone of littermate control B cells (CD19^Cre/+^ or N-WASP^Flox/Flox^) increased over time (Figure 2D). There was no significant BCR clustering and accumulation in the contact zone of B cells interacting with transferrin (Tf)-tethered lipid bilayer (Figure 2A,D), indicating that Fab′–anti-Ig aggregates reflects BCR clustering. In WKO and cNKO B cells, the TFI of labeled BCRs in the contact zone was significantly decreased compared to that of littermate control B cells (Figure 2D). While BCR accumulation in the contact zone of WKO and cNKO B cells was decreased to similar levels, the BCRs showed distinct distribution patterns. BCRs in the contact zone of WKO B cells formed a central cluster smaller than that of control B cells (Figure 2A), while in cNKO B cells they appeared punctate, failing to merge into a central cluster (Figure 2A). Treating MD4 B cells stimulated with membrane-associated HEL with the N-WASP inhibitor wiskostatin resulted in similar phenotypes as seen in cNKO B cells (Figure S1). The deletion of both *wasp* and *n-wasp* genes caused a further decrease in the BCR TFI in the B-cell contact zone, similar to the levels in unstimulated B cells (Figure 2A,D). Similarly, treating WKO B cells with the N-WASP inhibitor wiskostatin (Figure 2D) [51] or A20 lymphoma B cells with siRNAs targeted to WASP and N-WASP (Figure 2B,F) reduced the BCR TFI in the contact zone to levels similar to that in cDKO B cells. Furthermore, the BCR TFI in the contact zone of human B cells was decreased by wiskostatin treatment to levels similar to that of cNKO mouse B cells (Figure 2C,H).

We examined the effect of WASP and/or N-WASP KO on B-cell morphology by quantifying the changes in the contact area between B cells and Fab′–anti-Ig–tethered lipid bilayer, using interference reflection microscopy (IRM). In littermate control B cells, the contact area increased rapidly during the first 3 min, indicating cell spreading, and then decreased after reaching a maximal spread area, indicating cell contraction (Figure 2A,E). The change of WKO B-cell contact area over time was qualitatively similar to that of control B cells, but with a smaller magnitude of increase (Figure 2A,E). Both the kinetics and magnitude of the increase in the contact area of cDKO B cells were dramatically slower and smaller than those of control and WKO B cells (Figure 2A,E). Surprisingly, the contact area of cNKO B cells was significantly larger and decreased much later (7 min) than that of control B cells (Figure 2A,E). Again, treating WKO B cells with the N-WASP inhibitor wiskostatin (Figure 2E) or A20 B cells with WASP/N-WASP siRNAs (Figure 2B,G) reduced the B-cell contact area to sizes similar to that of cDKO B cells. Treating human primary B cells (Figure 2C,I) or HEL-stimulated mouse MD4 B cells (Figure S1) with wiskostatin caused an increase in B-cell spreading and a delay of B-cell contraction, similar to what we observed in cNKO B cells (Figure 2A,E).

Taken together, these results indicate that both WASP and N-WASP are indispensable for optimal BCR clustering and B-cell spreading, but N-WASP exhibits two opposing functions, supporting BCR clustering and B-cell spreading in the absence of WASP and promoting the coalescence of BCR microclusters into a central cluster and B-cell contraction in the presence of WASP. N-WASP exhibits similar functions in mouse and human primary B cells.

### BCR Signaling Is Enhanced and Prolonged in N-WASP KO B Cells

**Figure 3:**
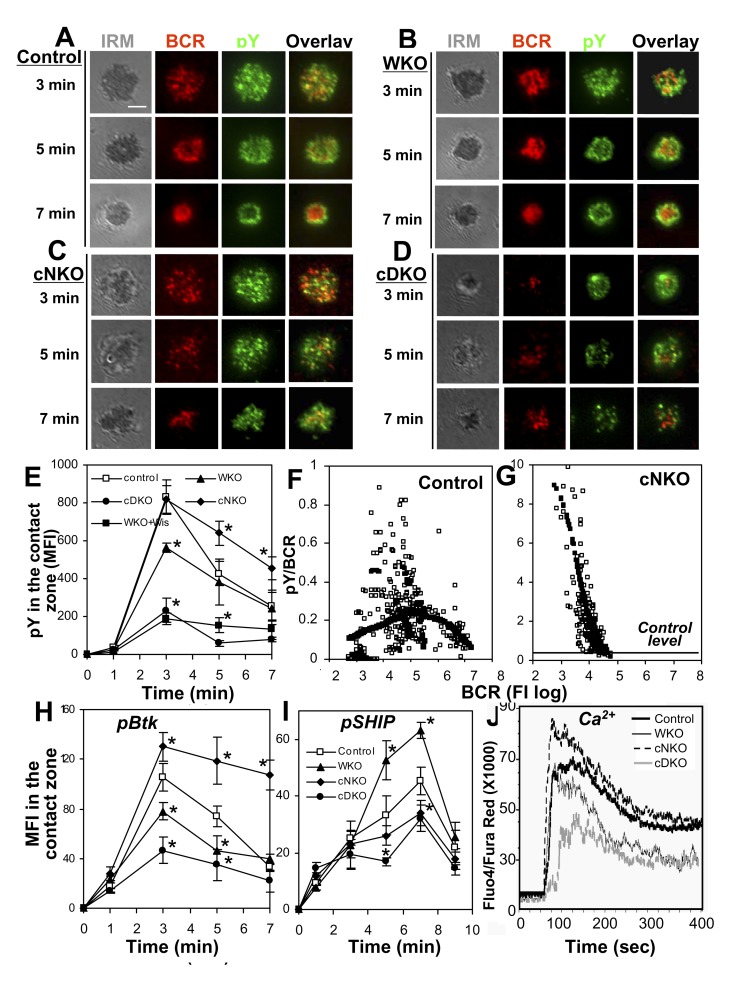
Differential effects of WASP and/or N-WASP KO on BCR signaling. (A–E) TIRFM and IRM analysis of pY staining in the contact zone of mouse splenic B cells incubated with membrane-tethered Fab′–anti-Ig. Shown are representative images (A–D) and the MFI (±SD) of pY in the B-cell contact zone (E) from three independent experiments. Bars, 2.5 µm. (F and G) The FIRs of pY to the BCR were plotted versus the TFI of the BCR in individual BCR clusters. Each open symbol represents a BCR cluster. The simulated values (solid symbol) were generated by LOSSE nonlinear regression using the Stat software. (H and I) TIRFM analysis of phosphorylated Btk (pBtk) and SHIP-1 (pSHIP) in the contact zone of mouse splenic B cells stimulated with membrane-tethered Fab′–anti-Ig. Shown are the average MFI (±SD) of pBtk and pSHIP in the B-cell contact zone from three independent experiments. *p<0.01, compared to littermate control B cells. (J) Ca^2+^ flux analysis of splenic B cells activated with soluble mB-Fab′–anti-Ig plus streptavidin using flow cytometry. Shown are representative results from three independent experiments.

The effects of WASP and/or N-WASP KO on BCR clustering and B-cell morphology suggest their involvement in BCR signaling. To test this hypothesis, we analyzed the impact of WASP and/or N-WASP KO on tyrosine phosphorylation (pY) at the cell surface in response to membrane-tethered Fab′–anti-Ig using TIRFM. Similar to what we have shown previously [21], pY was first detected at BCR microclusters at early times during the interaction of littermate control B cells with membrane-tethered Fab′–anti-Ig (∼3 min) and then at the outer edge of the BCR central cluster at later times (∼7 min) (Figure 3A). The MFI of pY staining rapidly increased upon BCR binding, peaked at 3 min, and then decreased (Figure 3E). The distribution and levels of pY in the contact zone of WKO B cells followed a qualitatively similar pattern as in control B cells, but the increasing magnitude of pY MFI in the contact zone of WKO B cells was significantly smaller than that of control B cells (Figure 3B,E). Double KO of WASP and N-WASP caused a further reduction in the levels of pY in the B-cell contact zone (Figure 3D,E). However, the pY staining in the contact zone of cNKO B cells remained punctate and colocalized with BCR clusters at 7 min (Figure 3C). The peak level of pY in the contact zone of cNKO B cells was similar to that of control B cells, but its attenuation was significantly delayed (Figure 3E). These results suggest that N-WASP is involved in both stimulation and attenuation of BCR signaling.

Our previous studies show a two-phase relationship between BCR clustering and signaling, where BCR aggregation into small clusters stimulates signaling activation, but the merger of small clusters into a central cluster leads to signaling attenuation at the cell surface [21]. The effects of cNKO on BCR clustering and B-cell contraction led us to hypothesize that N-WASP may regulate signaling via modulating the clustering of surface BCRs. To investigate this hypothesis, we determined the relative size of BCR clusters based on the TFI of BCR labeling in individual clusters and the relative signaling levels of BCR clusters based on the fluorescence intensity ratio (FIR) of the pY to the BCR in individual clusters. A nonparametric regression method, LOWESS, was used to examine the trend between the size and signaling level of BCR clusters. In littermate control B cells, the FIR of pY to BCR increased as the size of BCR clusters increased when their sizes were relatively small. After the clusters reached a certain size, the FIR decreased as the sizes of BCR clusters further increased (Figure 3F). Compared to control B cells, BCR clusters in the contact zone of cNKO B cells were limited to smaller sizes, and the FIR of pY to BCR in individual microclusters of cNKO B cells was much higher than that of control B cells (Figure 3G). These results show that the delayed attenuation of tyrosine phosphorylation in cNKO B cells is associated with the inhibition of the growth of BCR clusters, suggesting that N-WASP can down-regulate BCR signaling by promoting the growth and coalescence of BCR microclusters into the central cluster.

In order to confirm the roles of WASP and N-WASP in BCR signaling, we determined the effect of WASP and/or N-WASP KO on the phosphorylation of stimulatory kinase Btk (pBtk) and inhibitory phosphatase SHIP-1 (pSHIP-1) in response to membrane-tethered Fab′–anti-Ig by TIRFM and calcium flux in response to soluble Fab′–anti-Ig plus streptavidin by flow cytometry. Similar to the effect of WASP and/or N-WASP KO on pY, the MFI of pBtk was significantly reduced in the contact zone of WKO B cells and further reduced in that of cDKO B cells, compared to that of littermate control B cells (Figure 3H). However, the MFI of pBtk in the contact zone of cNKO B cells was not only significantly higher at the 3-min peak time, but the attenuation of pBtk was also significantly delayed, compared to that of control B cells (Figure 3H). In contrast, the MFI of pSHIP-1 in the contact zone was significantly increased in WKO B cells but reduced in cNKO B cells (Figure 3I). Double KO also caused a decrease in pSHIP-1 levels in the B-cell contact zone, but the magnitude of the decrease was similar to that in cNKO B cells (Figure 3I). Consistent with the changes in levels of pBtk and pSHIP-1 in the contact zone, the level of calcium influx was reduced in both WKO and cDKO B cells, with a much more dramatic reduction in cDKO than WKO B cells. In contrast, calcium influx was increased in cNKO B cells (Figure 3J).

These results collectively indicate critical roles for WASP and N-WASP in regulating BCR signaling in response to both membrane-associated and soluble antigen, and suggest dual functions for N-WASP in positive and negative signaling regulation.

### The Level of Autoantibody Is Elevated in Mice with B-Cell–Specific N-WASP Gene Deletion

**Figure 4:**
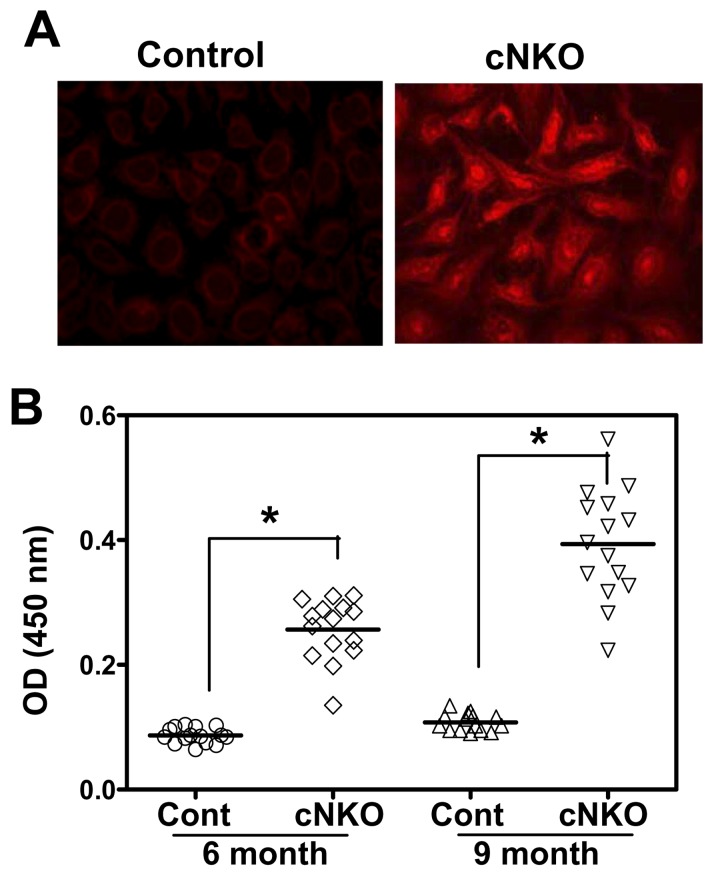
The serum levels of anti-nuclear and anti-dsDNA antibody are elevated in cNKO mice. (A) Representative images from immunofluorescence microscopic analysis of anti-nuclear antibody in the serum of littermate control and cNKO mice at 6 mo old (*n* = 4). (B) ELISA quantification of anti-dsDNA antibody in the serum of littermate control and cNKO mice at 6 and 9 mo old (*n* = 15). Each dot represents an individual mouse. *p<0.01.

The negative regulatory function of N-WASP in BCR activation implies a role for N-WASP in controlling B-cell self-tolerance. To investigate this possibility, we analyzed the serum levels of anti-nuclear and anti–double strand (ds) DNA antibody. Using an immunofluorescence test, we found that the sera of 50% cNKO mice were positive with anti-nuclear antibody (*n* = 4), compared to none of littermate control mice at 6 mo of age (Figure 4A). Consistent with this result, quantitative ELISA analysis detected an elevated level of anti-dsDNA antibody in the sera of cNKO mice, compared to littermate control mice at 6 and 9 mo old (Figure 4B). However, we did not detect any significant increase in the levels of anti-nuclear and anti-dsDNA antibody in the sera of WKO and cDKO mice compared to those of littermate control mice (Figure S4). Since the *n-wasp* gene deletion in cNKO mice is B-cell specific, our data indicate a critical and B-cell–intrinsic role for N-WASP in maintaining B-cell tolerance.

### WASP Promotes and N-WASP Inhibits F-actin Accumulation in the B-Cell Contact Zone

**Figure 5:**
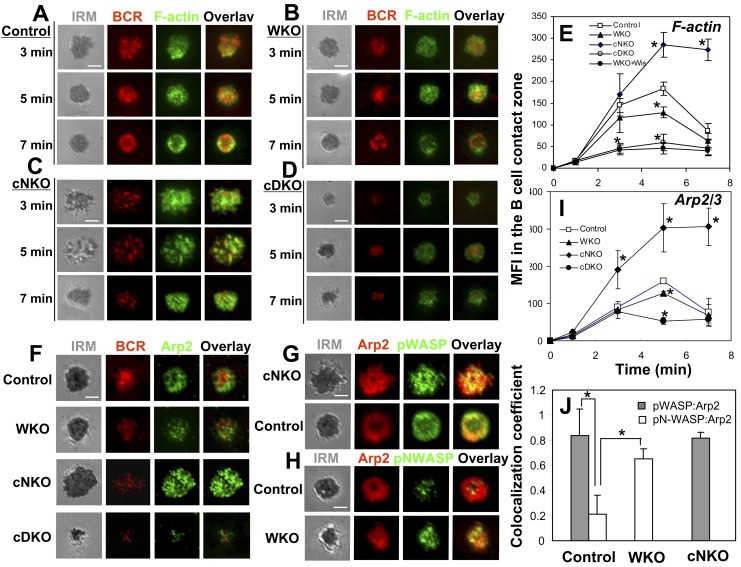
WASP promotes and N-WASP inhibits the F-actin accumulation in the B-cell contact zone. (A–E) TIRFM analysis of F-actin staining in the contact zone of splenic B cells incubated with membrane-tethered Fab′–anti-Ig. Shown are representative images (A–D) and the average MFI (±SD) of F-actin staining in the B-cell contact zone (E) from three independent experiments. (F and I) TIRFM analysis of Arp2 staining at the contact zone of splenic B cells incubated with membrane-tethered Fab′–anti-Ig. The MFI of Arp2 staining in the B-cell contact zone was quantified. (G, H, and J) TIRFM analysis of the spatial relationship of Arp2 with pWASP (G) or pN-WASP (H) in the contact zone of splenic B cells incubated with membrane-tethered Fab′–anti-Ig for 5 min. The colocalization coefficients between Arp2 and pWASP or pN-WASP staining were determined using Zeiss LSM software (J). Shown are representative images (F–H) and the average MFI (I) or colocalization coefficients (±SD) (J) from ∼50 individual cells of three independent experiments. Bars, 2.5 µm. *p<0.01, compared to B cells from littermate control mice.

Since the major function of WASP and N-WASP is to activate actin polymerization, we examined the effects of WASP and/or N-WASP KO on the distribution patterns and levels of F-actin in the B-cell contact zone using phalloidin staining and TIRFM analysis. When littermate control B cells spread on Fab′–anti-Ig–tethered lipid bilayer, F-actin formed smaller clusters throughout the contact zone and partially colocalized with BCR clusters. When B cells contracted and BCR microclusters coalesced into a central cluster, F-actin accumulated at the outer edge of the contact zone (Figure 5A). The level of F-actin in the contact zone of control B cells rapidly increased over time and peaked at 3–5 min when B-cell spreading reached maximal magnitude, followed by a significant reduction at 7 min when B cells contracted (Figure 2E and 5E). Gene KO of WASP or both WASP and N-WASP did not significantly change the distribution pattern of F-actin (Figure 5B,D) but reduced the level of F-actin accumulation in the B-cell contact zone (Figure 5E). The reduction was much more drastic in cDKO B cells and WKO B cells treated with the N-WASP inhibitor wiskostatin than in WKO B cells (Figure 5E). In contrast, the level of F-actin accumulation in the contact zone of cNKO B cells was significantly increased at 5 min and remained high at 7 min (Figure 5E). F-actin clusters formed in the contact zone of cNKO B cells appeared much more prominent than those in control B cells, and they displayed sustained colocalization with BCR clusters (Figure 5C) up to 7 min. These results suggest that N-WASP not only synergizes with WASP in generating and mobilizing F-actin to BCR clusters during signal activation, but is also critical for removing F-actin from the contact zone during B-cell contraction and surface signaling attenuation.

Since WASP and N-WASP share the same cellular function, activation of actin polymerization by binding to Arp2/3, we next asked how these two molecules exhibit opposing roles in actin remodeling during BCR activation. We analyzed the behavior of Arp2/3 in response to membrane-tethered Fab′–anti-Ig and its spatial relationship with pWASP and pN-WASP at the B-cell surface using TIRFM and Arp2-specific antibody. We found that Arp2 was readily recruited to the contact zone of littermate control B cells, and the timing of its recruitment, levels, and distribution patterns were similar to those of F-actin (Figure 5F,I). Consistent with the effect of WASP and/or N-WASP KO on F-actin accumulation, the MFI of Arp2 staining decreased slightly in the contact zone of WKO B cells and was significantly reduced in that of cDKO B cells, but increased in that of cNKO (Figure 5F,I). This result supports the notion that antigen-induced F-actin accumulation in the B-cell contact zone is mediated through the activation of Arp2/3 by WASP and N-WASP. The spatial relationship of Arp2 with active WASP and N-WASP were analyzed using Pearson correlation coefficients between the staining of Arp2 and pWASP or pN-WASP in the B-cell contact zone. The results showed that pWASP exhibited a significantly higher level of colocalization with Arp2 than pN-WASP in control B cells (Figure 5G,H,J). WASP KO caused a significant increase in the colocalization between pN-WASP and Arp2, close to the colocalization level of pWASP with Arp2 in control B cells (Figure 5H,J), but N-WASP KO did not change the level of colocalization between pWASP and Arp2 (Figure 5G,J). These data suggest that activated WASP predominately colocalizes with Arp2/3 while inhibiting the colocalization of active N-WASP with Arp2/3 at the B-cell surface.

### N-WASP Plays a Dominant Role in BCR Internalization

**Figure 6:**
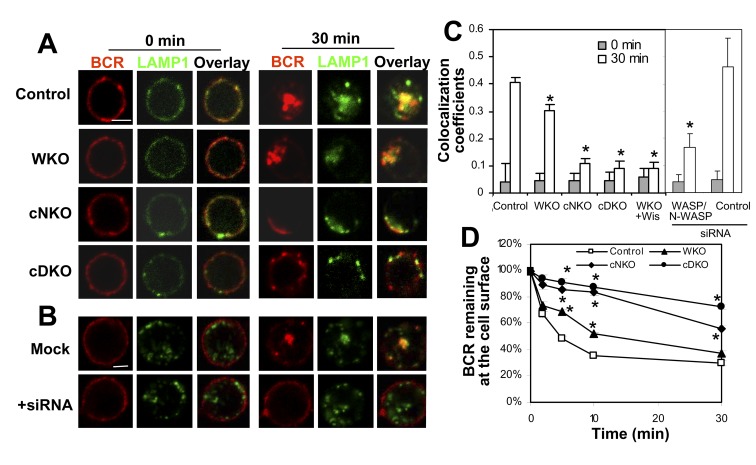
N-WASP plays a dominant role in BCR internalization. (A–C) Immunofluorescence microscopic analysis of BCR internalization. Colocalization coefficients between the surface-labeled BCR with LAMP-1 in splenic B cells (A and C) or A20 B cells transfected with WASP/N-WASP siRNA (B and C) were measured using the Zeiss LSM software. The surface BCR were labeled with AF546-mB-Fab′–anti-Ig plus soluble streptavidin and warmed to 37°C for 0 or 30 min. Shown are representative images (A and B) and the average correlation coefficients (±SD) (C) from >300 cells of three independent experiments. Bars, 2.5 µm. (D) Flow cytometry analysis of BCR internalization by quantifying the percentage of biotin-F(ab′)_2_–anti-Ig–labeled BCR remaining on the cell surface after the 37°C chase. Shown are the average percentages (±SD) from three independent experiments. *p≤0.05, compared to B cells from littermate control mice.

BCR activation induces receptor internalization, which attenuates receptor signaling by removing the receptor from surface signaling microdomains. Since BCR internalization requires actin reorganization [26], we investigated whether WASP and N-WASP are involved in this process. BCR internalization was evaluated qualitatively by the colocalization of surface-labeled BCRs with the late endosomal marker LAMP-1 using immunofluorescence microscopy and quantitatively by the amount of surface-labeled BCRs remaining at the cell surface after internalization using flow cytometry. In littermate control B cells, 70% of surface-labeled BCRs disappeared from the cell surface after internalization (Figure 6D), and they colocalized with LAMP-1–labeled late endosomes (Figure 6A,C) that coalesced in response to signaling [52]. In WKO B cells, the colocalization of surface-labeled BCRs with LAMP-1 was slightly decreased and the amount of the BCR remaining on the cell surface was increased (Figure 6A,C,D), indicating a reduction in BCR internalization. In cDKO B cells, the colocalization of the BCR with LAMP-1 was decreased from 0.4 to 0.1, and the amount of the BCRs remaining at the cell surface increased (∼80%), compared to those in littermate control B cells (∼30%), showing a dramatic reduction of BCR internalization (Figure 6A,C,D). Similarly, double knockdown of WASP and N-WASP by siRNA reduced the colocalization of the BCR with LAMP-1 in A20 cells (Figure 6B–C). Noticeably, cNKO B cells showed a similar level of reduction in the colocalization of BCR with LAMP-1 and a similar level of increase in surface BCRs as those observed in cDKO B cells, indicating that cNKO causes a decrease in BCR internalization similar in magnitude as cDKO. These results demonstrate that N-WASP plays a major role in this process.

### Mutual Regulation between WASP and N-WASP

**Figure 7:**
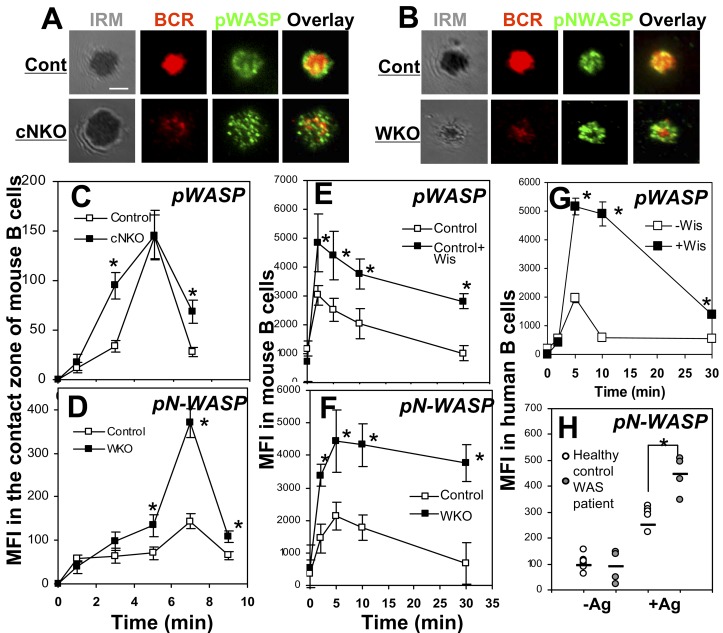
WASP and N-WASP negatively regulates each other. (A–D) TIRFM analysis of pWASP and pN-WASP in the contact zone of splenic B cells stimulated with membrane-tethered Fab′–anti-Ig (A and B). The MFI of pWASP (C) or pN-WASP (D) in the B-cell contact zone was quantified. (E–G) Flow cytometry analysis of the cellular MFI of pWASP or pN-WASP in splenic B cells from WKO and littermate control mice (F), and mouse splenic (E) and human PBMC B cells (G) that were treated with or without Wis and soluble mB-Fab′–anti-Ig plus streptavidin. (H) Flow cytometry analysis of the cellular MFI of pN-WASP in PBMC B cells from WAS patients and age-matched healthy donors that were incubated with or without soluble mB-Fab′–anti-Ig plus streptavidin for 2 min. Shown are representative images at 7 min and the average MFI (±SD) from three independent experiments. Bars, 2.5 µm. * *p*<0.01, compared to B cells from wt or littermate control mice, without Wis treatment or healthy donors.

The involvement of both WASP and N-WASP in BCR activation suggests a functional coordination between these two proteins. To understand their functional relationship, we compared the activation levels and kinetics of one of the two proteins in the presence and absence of the other using TIRFM and flow cytometry. Both the MFI of pWASP in the contact zone of cNKO B cells (Figure 7A,C) and the cellular MFI of wiskostatin-treated mouse B cells (Figure 7E) were increased compared to those in untreated control B cells. Conversely, the pN-WASP levels in the contact zone of WKO B cells (Figure 7B,D) and in WKO B cells (Figure 7F) were significantly higher than those in littermate control B cells. In both cases, the time taken for pN-WASP to peak was not changed. While the levels of pN-WASP and pWASP were changed, the overall protein levels of N-WASP and WASP did not change in WKO and cNKO B cells (Figure S5), indicating that the increased phosphorylation level is not due to an increase in protein expression. Consistent with the data from mouse models, treating human B cells from healthy subjects with the N-WASP inhibitor wiskostatin resulted in an increase in the cellular level of pWASP (Figure 7G). Furthermore, the level of pN-WASP was increased in PBMC B cells from WAS patients that did not express or expressed low levels of WASP, compared to that of healthy human controls (Figure 7H). These results indicate that WASP and N-WASP negatively regulate each other during BCR activation in both mouse and human B cells.

### The Activation of WASP and N-WASP Is Inversely Regulated by BCR Signaling

**Figure 8:**
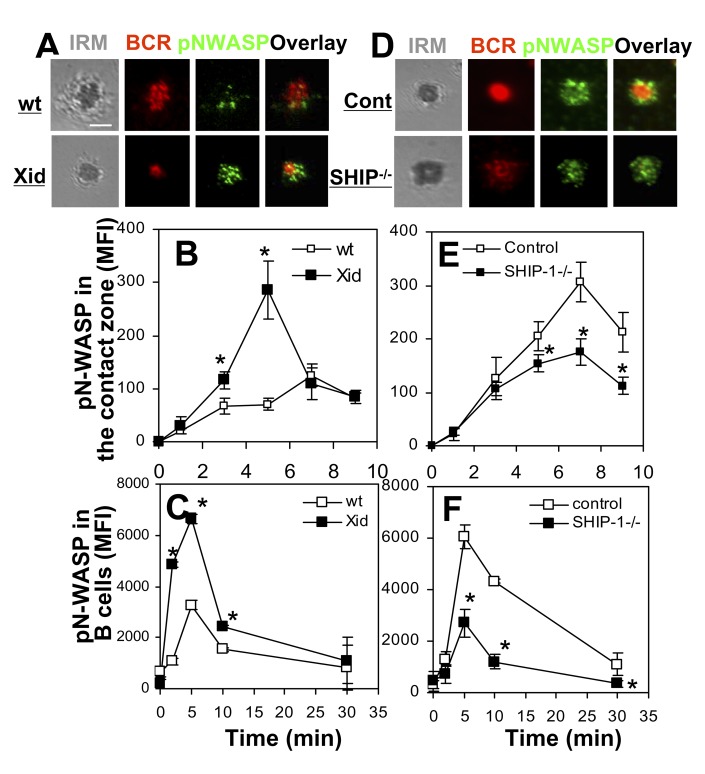
WASP and N-WASP are inversely regulated by Btk and SHIP-1. (A–B and D–E) TIRFM analysis of pN-WASP in the contact zone of splenic B cells from wt, xid, control, and B-cell–specific SHIP-1^−/−^ mice that were incubated with membrane-tethered Fab′–anti-Ig. The MFI of pN-WASP in the B-cell contact zone was determined (B and E). (C and F) Flow cytometry analysis of the cellular MFI of pN-WASP in splenic B cells incubated with soluble mB-Fab′–anti-Ig plus streptavidin. Shown are representative images at 7 min and the average MFI (±SD) from three independent experiments. Bars, 2.5 µm. * *p*<0.01, compared to B cells from wt or littermate control mice.

The activation of WASP and N-WASP in response to BCR cross-linking suggests that BCR signaling triggers their activation. We have previously shown that Btk is responsible for activating WASP, while SHIP-1 suppresses WASP activation by inhibiting Btk [21]. To investigate whether N-WASP activation is controlled in the same manner as WASP, we determined the effect of Btk or SHIP-1 deficiency on antigen-triggered phosphorylation of N-WASP using xid mice where the PH domain of Btk contains a point mutation that blocks Btk activation [53] and B-cell–specific SHIP-1 KO mice [21],[54]. In sharp contrast with the effect of Btk and SHIP-1 deficiency on WASP activation, the MFI of pN-WASP was increased in the B-cell contact zone and B cells from xid mice (Figure 8A–C), while it was decreased in the B-cell contact zone and B cells from SHIP-1 KO mice (Figure 8D–F). These results suggest that the activation of WASP and N-WASP is regulated inversely by BCR signaling: Btk, which activates WASP, suppresses N-WASP activation, while SHIP-1, which inhibits the activation of Btk and WASP, promotes N-WASP activation.

## Discussion

In this study, we demonstrate that in addition to the overlapping function with WASP in signaling activation, N-WASP plays a unique role in the down-regulation of BCR signaling at the cell surface. This is shown by enhanced and/or prolonged tyrosine and Btk phosphorylation, increased calcium influx, and reduced SHIP-1 phosphorylation in cNKO B cells. Importantly, the enhanced signaling in response to in vitro antigenic stimulation is associated with elevated levels of anti-nuclear and anti-dsDNA autoantibodies in the serum of cNKO mice. Since N-WASP is exclusively deleted from B cells, the increased autoantibody and BCR signaling is the result of B-cell–intrinsic defects. These results indicate that N-WASP–mediated signal attenuation of surface BCRs is critical for the maintenance of B-cell self-tolerance.

Our studies find that there are more severe defects in BCR clustering, B-cell spreading, and signal activation in cDKO B cells than in WKO B cells, indicating overlapping functions between N-WASP and WASP in these processes. This is consistent with their shared cellular function in promoting actin polymerization. However, WASP KO alone results in significant decreases in these processes, even though they are much less severe than those in cDKO B cells. These results suggest that N-WASP is unable to completely compensate for WASP in WKO B cells.

Our data further show that the inhibition of the attenuation of BCR surface signaling is concurrent with increases in B-cell spreading, delays in B-cell contraction and the coalescence of BCR microclusters into the central cluster, and a blockage of BCR internalization in cNKO B cells. These results suggest that N-WASP promotes signaling attenuation via modulating BCR clustering, B-cell morphology, and BCR internalization. Similar to what we have previously shown in SHIP-1^−/−^ B cells [21], BCR clusters in cNKO B cells remain smaller in size and exhibit much higher levels of tyrosine phosphorylation than those in littermate control B cells, while cNKO B cell contraction is delayed in comparison with littermate control B cells. These data collectively demonstrate that B-cell contraction, which can provide a force for the merger of small BCR microclusters into polarized central clusters, is an important mechanism for down-regulating BCR signaling at the cell surface. The exact mechanism by which the size of BCR clusters regulates receptor signaling is unknown. Our results show that the levels of pBtk and pSHIP-1 are increased and decreased, respectively, in the contact zone of cNKO B cells, suggesting an association of the activation of positive and negative signaling molecules with the size of BCR clusters and N-WASP expression. N-WASP has been suggested to have a role in regulating the cellular location and activation of SHIP [55]. The dominant role of N-WASP in BCR internalization enables the removal of antigen–BCR complexes from the cell surface, which leads to both surface signaling attenuation and antigen presentation that provides another layer of control over B-cell activation by T cells.

While N-WASP has both an overlapping role with WASP in signaling activation and a unique role in signaling down-regulation in B cells, we demonstrate here that N-WASP exhibits the two opposing functions under different circumstances. N-WASP predominantly displays its function for signaling down-regulation in B cells that have normal expression of WASP, since cNKO B cells have no defects in B-cell spreading and signaling activation. However, it switches to signaling activation function in B cells lacking WASP expression, since cDKO B cells have more severe signaling defects than WKO B cells. The different functionalities of N-WASP in the presence or absence of WASP expression suggests that WASP is involved in the functional switch of N-WASP. Indeed, we found that in littermate control B cells, the pWASP level increases first, and the pN-WASP level does not peak until pWASP returns near to its basal level. In the absence of WASP, the pN-WASP level rises earlier and significantly higher in WKO B cells than that in littermate control B cells. Conversely, in the absence of N-WASP, the pWASP level increases earlier and is sustained longer in cNKO B cells than that in littermate control B cells. Moreover, we found similar results in human B cells from healthy subjects and WAS patients. These results indicate that WASP and N-WASP mutually suppress each other for activation in both human and mouse B cells.

Previous studies have shown that WASP and N-WASP share the same activation mechanisms, including the interaction with the GTPase Cdc42 or Rac via the GBD domain and PI(4,5)P_2_ via the PH domain, and tyrosine and serine phosphorylation [43],[46],[56]. We have previously shown that Btk is responsible for the activation of WASP in B cells by activating guanine nucleotide exchange factor Vav, increasing PI(4,5)P_2_, and inducing WASP phosphorylation [50]. A surprising finding of this study is that N-WASP is not activated by the same pathway as WASP, since the level of pN-WASP is increased rather than decreased in Btk-deficient B cells and is decreased rather than increased in SHIP-1^−/−^ B cell as seen for pWASP. This suggests that SHIP-1 instead of Btk is involved in N-WASP activation. During BCR activation, Btk is activated before SHIP-1 [2],[10],[57], which provides an explanation for the activation of WASP before N-WASP. SHIP-1 inhibits Btk activation via dephosphorylating PI(3,4,5)P_3_, the docking site of Btk at the plasma membrane [58], consequently suppressing WASP activation. The molecular mechanisms by which SHIP-1 activates N-WASP are unknown. Based on our data, we postulate that releasing N-WASP from WASP suppression by inhibiting Btk-dependent WASP activation is one possible mechanism for SHIP-1 to facilitate N-WASP activation. In addition, SHIP-1 potentially regulates the location and activation of N-WASP via the signaling adaptor protein Grb2. Grb2 has been reported to bind both N-WASP and the SHIP adaptor protein Dok [42],[59],[60]. Further, the product of SHIP-1–mediated hydrolysis PtdIn(3,4)P_2_ has been shown to regulate N-WASP by recruiting Tks5-Grb2 scaffold [61]. SHIP may also indirectly regulate the subcellular location and activity of kinases that phosphorylate N-WASP. These possible mechanisms remain to be further examined.

Studies accumulated from the last decade have clearly demonstrated that WASP and N-WASP share the same cellular function: stimulating actin polymerization by activating Arp2/3 [44],[46],[62]. This raises the question of how these two proteins could possibly have opposing roles in B-cell morphology and BCR clustering and activation. It should be noted that almost all of the studies so far have examined the cellular function of WASP and N-WASP individually in the absence of their homolog. Consistent with these studies, we found that WASP and N-WASP appear to play a similar role in actin accumulation at the B-cell contact zone, shown by a smaller decrease in the level of F-actin in the contact zone of WKO B cells than that of cNKO B cells. Surprisingly, N-WASP KO alone causes a significant and sustained increase in the level of F-actin in the B-cell contact zone, which is inconsistent with the current dogma that N-WASP functions as an actin-nucleation–promoting but not inhibitory factor. Our examination of the spatial relationship of activated WASP and N-WASP with Arp2/3 shows that pWASP has a significantly higher level of colocalization with Arp2 than pN-WASP, and that WASP KO increases the colocalization between pN-WASP and Arp2, but N-WASP KO does not lead to further increases in the colocalization of pWASP with Arp2. These results suggest a competition between WASP and N-WASP for binding to Arp2/3 complexes. Since WASP is activated and recruited to the B-cell contact zone by Btk first, this allows WASP to win the competition for binding to Arp2/3, consequently competitively inhibiting the binding of N-WASP to Arp2/3.

How N-WASP reduces the amount of F-actin in the B-cell contact zone and inhibits B-cell spreading is an interesting question. When Takenawa's group discovered N-WASP, N-WASP was identified as an actin depolymerizing protein since the VCA domain of N-WASP was capable of depolymerizing F-actin in vitro in the absence of Arp2/3 [42]. This raises the possibility that the VCA domain of active N-WASP, when it is not bound by Arp2/3 as it loses the competition to activated WASP, can depolymerize F-actin at the B-cell contact zone. In addition, N-WASP has a unique function in tethering F-actin to budding and moving vesicles [63],[64]. We have previously demonstrated that the membrane fission step of BCR endocytosis requires actin [26]. The dominant role of N-WASP in BCR internalization implicates that N-WASP is responsible for this actin-dependent step, probably by recruiting F-actin from the B-cell contact zone to and/or activating actin polymerization at budding vesicles containing BCRs [65],[66]. Furthermore, the involvement of N-WASP in SHIP-1 activation reported here and the capability of N-WASP to interact with BCR adaptor proteins, such as Grb2 [42], suggest that N-WASP may inhibit actin polymerization and B-cell spreading by facilitating or enhancing the activation of inhibitory signaling molecules.

WASP deficiency due to gene mutations causes an X-linked immune disorder, exhibiting immune deficiency, autoimmunity, and lymphoma [30],[32],[36]. While defects in other immune cells contribute to the disease, recent studies have demonstrated that B-cell–intrinsic defects are critical for the development of autoimmunity in mouse models [39],[41]. Here we show that WASP and N-WASP behave similarly in mouse and human B cells, including the sequential activation of N-WASP and WASP and the mutual regulation between the two. The B-cell–intrinsic roles of N-WASP in the down-regulation of BCR signaling and B-cell tolerance demonstrated here suggest a critical contribution of N-WASP to disease development. It is possible that without the competitive inhibition of WASP, the signaling promoting function of N-WASP is enhanced and its signaling attenuation function is reduced, leading to deregulation of BCR and B-cell activation. This hypothesis will be pursued in our future studies.

It is well known that the genetic background influences the characteristics of the mouse immune system and the susceptibility of mice to autoimmune diseases [67],[68]. Due to triple genetic manipulations, the mouse models used in this study were comprised of a mixed background of C57BL/6 and 129Sv. Given our breeding strategy (described in the Materials and Methods section), the CD19^Cre/Cre^ mice on a C57BL/6 background bring B6 genes into WASP^−/−^ and N-WASP^Flox/Flox^ mice on a 129Sv background. The CD19 Cre allele from CD19^Cre/+^ mice generates a systematic bias for B6 mouse chromosome 7, where CD19 is located, while *n-wasp* and *wasp* genes are located in chromosome 6 and X chromosome of mice, respectively. We utilized CD19^Cre/+^ mice for the final crossing step, which enables us to generate CD19^+/+^ littermates with similar numbers of B6 alleles as CD19^Cre/+^ littermates, thereby providing littermate controls. By using more than 15 sets of littermate controls to compare with WKO, cNKO, and cDKO mice, we found a consistent and significant increase in the level of serum autoantibody in cNKO mice as well as increased spreading of cNKO B cells. While CD19^Cre/+^ C57BL/6 mice would provide an additional control for ruling out any contribution of genetic background to the results, our data with 15–18 littermate controls improves confidence that the dosage of B6 genes is not biasing the results regarding the negative regulation mediated by N-WASP.

**Figure 9:**
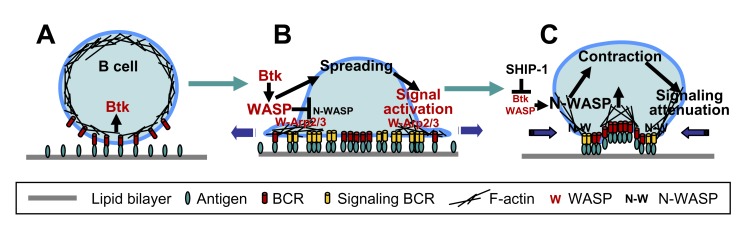
A working model for the coordination of N-WASP with WASP in the regulation of BCR signaling. (A) Antigen binding to the BCR induces an early activation of Btk. (B) Activated Btk in turn activates and translocates WASP to the cell surface. Activated WASP (W) stimulates actin polymerization and reorganization by binding to Arp2/3 and suppresses N-WASP activation, which drives B-cell spreading and facilitates BCR clustering and signaling. (C) The activation of SHIP-1 induced by the BCR after initial signaling inhibits Btk and WASP activation, consequently releasing N-WASP from WASP suppression. Active N-WASP decreases actin accumulation at the B-cell contact zone, which enables B-cell contraction and promotes the coalescence of BCR microclusters into a central cluster and BCR internalization. The formation of BCR central clusters and BCR endocytosis lead to the down-regulation of BCR signaling at the cell surface.

Taking the results of this study and previous studies together enables us to propose a working model for the functional coordination of WASP and N-WASP during BCR activation (Figure 9). Antigen binding to the BCR induces an early activation of Btk (Figure 9A) that in turn activates and translocates WASP to the cell surface. Activated WASP stimulates actin polymerization by binding to Arp2/3, which modulates BCR lateral mobility and drives B-cell spreading. Together, these facilitate BCR clustering and signaling (Figure 9B). The activation of SHIP-1 after the initial signaling inhibits Btk activation, which decreases the level of active WASP and releases N-WASP from the suppression of WASP. The activated N-WASP reduces the surface level of F-actin probably by depolymerizing and/or transferring F-actin to BCR containing budding vesicles. Reductions in F-actin at the B-cell contact zone allow B-cells to contract, which promotes the coalescence of BCR microclusters into a central cluster. Tethering F-actin to endocytosing vesicles by N-WASP is essential for the endocytosis of antigen–BCR complexes. The formation of BCR central clusters and BCR endocytosis lead to the down-regulation of BCR signaling at the cell surface (Figure 9C). While molecular details of this working model require further investigation, this study reveals a novel function of N-WASP in the down-regulation of BCR signaling and a unique functional coordination between WASP and N-WASP during receptor signaling.

## Materials and Methods

All experiments involving human and mouse samples were performed using protocols approved by the authors' institutional review board or institutional animal care and usage committee and following institutional and NIH guidelines and regulations.

### Mice and Cells

Wild-type (wt) (CBA/CaJ), xid (CBA/CaHNBtkxid/J), MD4 Ig transgenic, and CD19^Cre/+^ (B6.129P2(C)-Cd19^tm1(cre)Cgn^/J) mice were purchased from Jackson Laboratories (Bar Harbor, ME). B-cell–specific SHIP-1 knockout mice (CD19^Cre/+^ SHIP-1^Flox/Flox^) were kindly provided by Dr. Silvia Bolland at NIH [21],[54]. WASP knockout (WASP^−/−^ CD19^+/+^ N-WASP^Flox/Flox^, WKO), B-cell–specific N-WASP knockout (CD19^Cre/+^ N-WASP^Flox/Flox^, cNKO), WASP and N-WASP double conditional knockout (WASP^−/−^ CD19^Cre/+^ N-WASP^Flox/Flox^, cDKO) mice were bred as previously described [38]. These mice are generated by breeding WKO mice on a 129Sv background, N-WASP^Flox/Flox^ mice on a 129Sv background, and CD19^Cre/Cre^ mice on a C57BL/6 background. The data were generated from breeding littermates of CD19^Cre/+^ N-WASP^Flox/Flox^ with CD19^Cre/+^ N-WASP^Flox/Flox^ for littermate control mice (CD19^+/+^ N-WASP^Flox/Flox^) and cNKO mice (CD19^Cre/+^ N-WASP^Flox/Flox^) and of WASP^−/−^ CD19^Cre/+^ N-WASP^Flox/Flox^ with WASP^−/−^ CD19^Cre/+^ N-WASP^Flox/Flox^ for WKO (WASP^−/−^ CD19^+/+^ N-WASP^Flox/Flox^) and cDKO (WASP^−/−^ CD19^Cre/+^ N-WASP^Flox/Flox^). The littermates of cNKO breeding that have neither WASP nor N-WASP deficiency were used as controls (CD19^+/+^N-WASP^Flox/Flox^). B-cell lymphoma A20 IIA1.6 cells (H-2d, IgG2a^+^, FcγIIBR^−^) were cultured and splenic B cells were isolated as previously described [50]. Human peripheral blood mononuclear cells (PBMCs) were collected from WAS patients, age-matched healthy controls, and healthy adults. B cells were isolated from human PBMCs using a Miltenyi Human B-cell negative selection kit by AutoMACS (Miltenyi Biotec Inc., Auburn, CA).

### Preparation of Antigen-tethered Planar Lipid Bilayers

Mono-biotinylated Fab′ fragment of anti-mouse or human IgM+G antibody (mB-Fab′–anti-Ig) was generated from the F(ab′)_2_ fragment (Jackson ImmunoResearch, West Grove, PA) using a published protocol [69]. The planar lipid bilayer was prepared as described previously [70],[71]. Liposomes were made by sonicating 1,2-dioleoyl-sn-Glycero-3-phosphocholine and 1,2-dioleoyl-sn-Glycero-3-phosphoethanolamine-cap-biotin (Avanti Polar Lipids, Alabaster, AL) in a 100∶1 molar ratio in PBS. Coverslip chambers (Nalge Nunc International, Rochester, NY) were incubated with the liposomes before coating with 1 µg/ml streptavidin (Jackson ImmunoResearch) and 2 µg/ml AF546–mB-Fab′–anti-Ig mixed with 8 µg/ml mB-Fab′–anti-Ig antibody. For a nonantigen control, surface BCRs were labeled with AF546-Fab–anti-Ig (2 µg/ml). The labeled B cells were then incubated with biotinylated holo-transferrin (Tf; 16 µg/ml, Sigma, St. Louis, MO) tethered to lipid bilayers by streptavidin.

### Total Internal Reflection Fluorescence Microscopy

Images were acquired using a Nikon TE2000-PFS microscope equipped with a 60×, NA 1.49 Apochromat TIRF objective, a Coolsnap HQ2 CCD camera (Roper Scientific), and two solid-state lasers of wavelength 491 and 561 nm. Interference refection images (IRM), AF488, and AF546 were acquired sequentially. B cells were incubated with AF546–mB-Fab′–anti-Ig–tethered lipid bilayers at 37°C, and then were then fixed with 4% paraformaldehyde, permeabilized with 0.05% saponin, and stained for phosphotyrosine (Millipore), phosphorylated Btk (BD Bioscience, San Jose, CA), SHIP-1 (Cell Signaling Technology, Inc., Danvers, MA), WASP (S483/S484 or Y290) (Bethyl Laboratory, Inc., Montgomery, TX or Abcam, Cambridge, MA), N-WASP (Y256, Millipore), and AF488-phalloidin. The B-cell contact area was determined based on IRM images using MATLAB software (The MathWorks, Inc. Natick, MA). The total and mean fluorescence intensity in the B-cell contact zone was determined using Andor iQ software (Andor Technology, Belfast, UK). Background fluorescence generated by antigen or secondary antibody was subtracted. For each set of data, >50 individual cells from three independent experiments were analyzed.

### Calcium Flux

The intracellular calcium flux was measured by flow cytometry using the calcium-sensitive dyes Fluo4 AM and Fura Red (Invitrogen) using manufacturer-recommended protocols. The relative levels of intracellular calcium were determined by a ratio of Fluo4 to Fura Red emission using FlowJo software (Tree Star, Inc., Ashland, OR).

### BCR Internalization

#### Flow cytometry

B cells were incubated with biotinylated F(ab′)_2_-goat anti-mouse IgG+M (10 µg/ml; Jackson ImmunoResearch) at 4°C and chased at 37°C [72]. Biotin-F(ab′)_2_–anti-IgG+M left on the cell surface after the chase was stained with PE-streptavidin and quantified using a flow cytometer. The data were presented as percentages of the cell-surface–associated biotin-F(ab′)_2_–anti-IgG+M at time 0.

#### Immunofluorescence microscopy

B cells that were labeled and cross-linked as described above were chased for 30 min [72]. The cells were fixed, permeabilized, stained for LAMP-1 (1D4B; ATCC), and analyzed using a confocal microscope (Zeiss LSM 710). Pearson correlation coefficients were determined using LSM 710 software [73].

### Flow Cytometry Analysis

B cells were incubated with FcR blocking antibodies (BD) and then with PE-Cy7–anti-CD19 (BD) at 4°C. B cells were activated with F(ab′)_2_–anti-Ig(M+G) (10 µg/ml, Jackson ImmunoResearch) at 37°C. The cells were fixed, permeabilized, and stained with anti-phosphorylated WASP (S483/S484) and N-WASP (Y256) antibodies for mouse B cells, anti-phosphorylated WASP (Y290) (Abcam, Cambridge, MA) and N-WASP (Y256, Millipore) antibodies for human B cells, and anti-WASP or N-WASP (Santa Cruz, CA) antibody for total protein. Stained cells were analyzed by a BD FACS Canto. Anti-phosphorylated WASP antibody showed no significant staining in B cells from WKO mice and anti-phosphorylated N-WASP antibody showed no staining in B cells from cNKO mice (Figure S2), indicating that there is no cross-reactivity of these two antibodies between phosphorylated WASP and N-WASP.

### Inhibitors

B cells were pretreated with wiskostatin B (10 µM, EMD Bioscience, Gibbstown, NJ) for 1 h at 37°C. The inhibitor was also included in the incubation media.

### Serological Analysis

Anti-nuclear antibody in sera was tested using the ANA slide test kit from MBL-Bion (Des Plaines, IL). The serum levels of anti-dsDNA antibody were quantified by ELISA using a published protocol [41].

### Reverse Transcriptase-PCR(RT-PCR) and Western Blot

B cells were incubated with FcγR blocking antibodies (BD) and then with PE-Cy7-anti-CD19 (BD) at 4°C. CD19 positive cells were sorted with BD FACS Aria II, and mRNAs were extracted using Trizol (Invitrogen). RT-PCR was carried out using the SuperScript III One-Step RT-PCR System (Invitrogen). WASP and N-WASP were amplified using specific primers from Santa Cruz Biotechnology, and β-tubulin was amplified as a control. Lysates were generated from sorted CD19 positive cells and analyzed by SDS-PAGE and Western blotting. Blots were probed with WASP- (Cell signaling) and N-WASP–specific antibodies (Santa Cruz), and β-tubulin–specific antibody (Sigma) as loading controls.

### Statistical Analysis

Statistical significance was assessed by the two-tailed student's *t* test using Prism software (GraphPad Software, San Diego, CA).

## Supporting Information

Figure S1 **BCR clustering and cell spreading in MD4 B cells induced by hen egg lysozyme tethered to lipid bilayers.** B cells from MD4 mice were pretreated with DMSO or wiskostatin (Wis), stimulated with fluorescently labeled and biotinylated hen egg lysozyme (HEL) tethered to lipid bilayers for indicated times, and analyzed by TIRFM and IRM. Shown are representative images at 7 min (A). Bar, 2.5 µm. The average TFI (±SD) of HEL in the B-cell contact zone (B) and the average value (±SD) of B-cell contact area (C) were determined using TIRFM and IRM images from >100 individual cells of three independent experiments. *p<0.01, compared to B cells treated with DMSO.
(TIF)

Figure S2 **Antibodies specific for phosphorylated WASP or phosphorylated N-WASP do not cross-react with each other.** Splenic B cells from wt, WKO, and cNKO mice were stimulated with F(ab′)_2_–anti-mouse IgM+G (10 µg/ml) at 37°C for 2 or 5 min, fixed, permeabilized, stained for pWASP, pN-WASP, and isotype control (Iso Con), and analyzed using flow cytometry. Shown are the average MFI (±SD). *n* = 3.
(TIF)

Figure S3 **The mRNA and protein expression of WASP and N-WASP in B cells from WKO, cNKO, and cDKO mice.** B cells sorted from splenocytes of littermate control, WKO, cNKO, and cDKO mice. (A) The mRNAs were extracted, and RT-PCR was performed. WASP and N-WASP were amplified by PCR using specific primers. (B) Cells were lysed and analyzed by SDS-PAGE and Western blot that were blotted with WASP- and N-WASP–specific antibodies.
(TIF)

Figure S4 **The serum levels of anti-nuclear and anti-dsDNA antibody are not significantly increased in WKO and cDKO mice.** (A) Representative images from immunofluorescence microscopic analysis of anti-nuclear antibody in the serum of littermate control, WKO, and cDKO mice of 6 mo old (*n* = 4). (B) ELISA quantification of anti-dsDNA antibody in the serum of littermate control, WKO, and cDKO mice at 6 and 9 mo old. Each dot represents an individual mouse. *n* = 15.
(TIF)

Figure S5 **WASP or N-WASP gene knockout does not affect the overall expression levels of N-WASP and WASP.** Splenic B cells from wt, littermate control, WKO, and cNKO mice were fixed, permeabilized, stained for WASP and N-WASP, and analyzed using flow cytometry. The average MFI (±SD) was generated from three independent experiments.
(TIF)

## Abbreviations

AF456–mB-Fab′–anti-Ig: Alexa Fluor 456–labeled, monobiotinylated Fab′ fragment of anti-mouse or human IgG+M
BCR: B-cell receptor
Btk: Bruton's tyrosine kinase
cDKO: double knockout
cNKO: B-cell–specific N-WASP knockout
FIR: fluorescence intensity ratio
HEL: hen egg lysozyme
KO: knockout
MFI: mean fluorescence intensity
N-WASP: Neuronal Wiskott-Aldrich syndrome proteins
PBMC: peripheral blood mononuclear cells
pBtk: phosphorylated Btk
PI(3,4,5)P_3_: phosphatidylinositol-3,4,5-triphosphate
PLCγ2: phospholipase Cγ2
pN-WASP: phosphorylated N-WASP
pSHIP: phosphorylated SHIP
pWASP: phosphorylated WASP
pY: phosphotyrosine
SHIP: Src-homology 2-containing inositol 5-phosphatase
TFI: total fluorescence intensity
TLR: Toll-like receptor
WASP: Wiskott-Aldrich syndrome proteins
WKO: WASP knockout
