# Supplementary figures and images for: N-WASP Is Essential for the Negative Regulation of B Cell Receptor Signaling
Source: PLoS Biol. 2013 Nov 5;11(11):e1001704. doi: 10.1371/journal.pbio.1001704 (PMC3818172; doi:10.1371/journal.pbio.1001704)

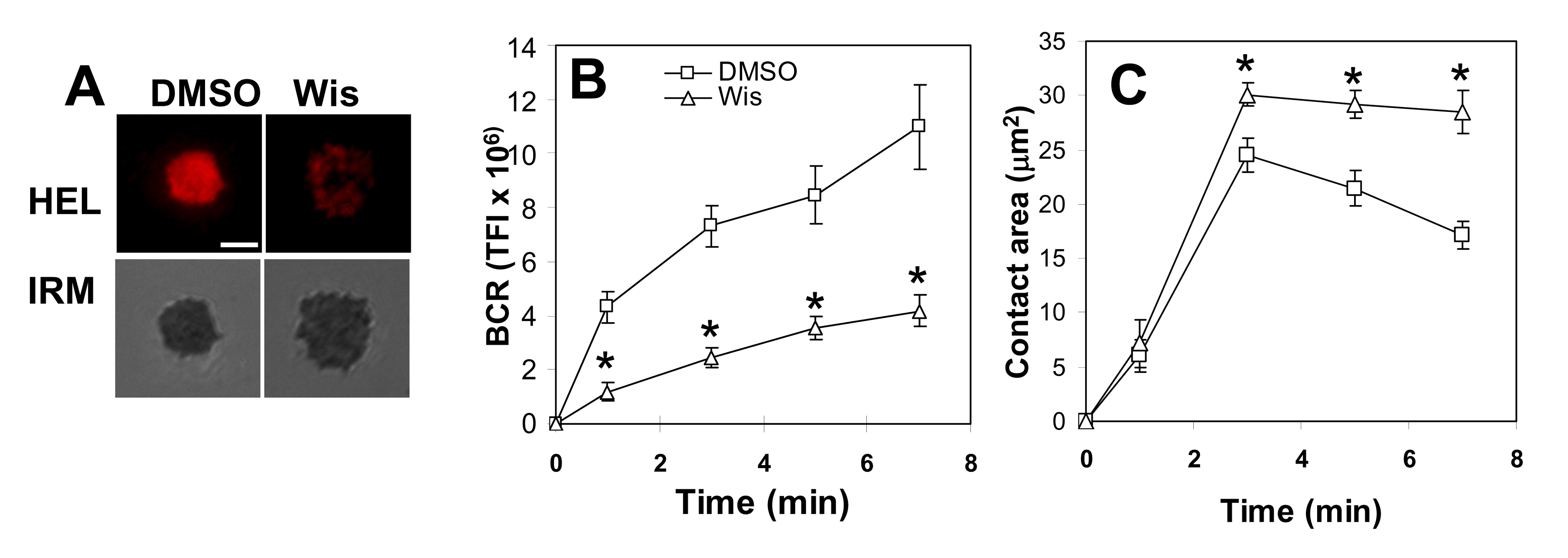

Supplement: Figure S1 — BCR clustering and cell spreading in MD4 B cells induced by hen egg lysozyme tethered to lipid bilayers. B cells from MD4 mice were pretreated with DMSO or wiskostatin (Wis), stimulated with fluorescently labeled and biotinylated hen egg lysozyme (HEL) tethered to lipid bilayers for indicated times, and analyzed by TIRFM and IRM. Shown are representative images at 7 min (A). Bar, 2.5 µm. The average TFI (±SD) of HEL in the B-cell contact zone (B) and the average value (±SD) of B-cell contact area (C) were determined using TIRFM and IRM images from >100 individual cells of three independent experiments. *p<0.01, compared to B cells treated with DMSO. (TIF) [file pbio.1001704.s001.tif]

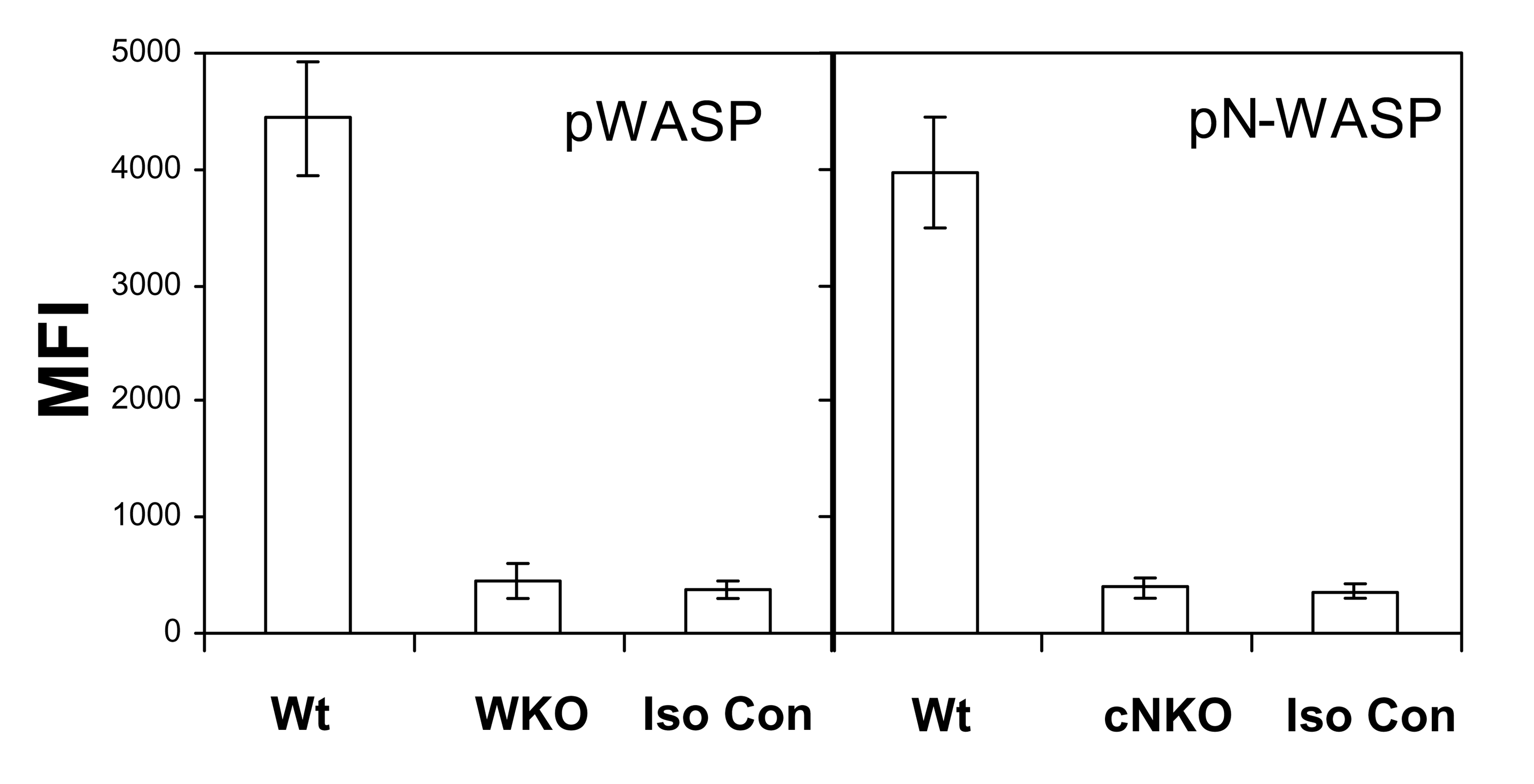

Supplement: Figure S2 — Antibodies specific for phosphorylated WASP or phosphorylated N-WASP do not cross-react with each other. Splenic B cells from wt, WKO, and cNKO mice were stimulated with F(ab′)2–anti-mouse IgM+G (10 µg/ml) at 37°C for 2 or 5 min, fixed, permeabilized, stained for pWASP, pN-WASP, and isotype control (Iso Con), and analyzed using flow cytometry. Shown are the average MFI (±SD). n = 3. (TIF) [file pbio.1001704.s002.tif]

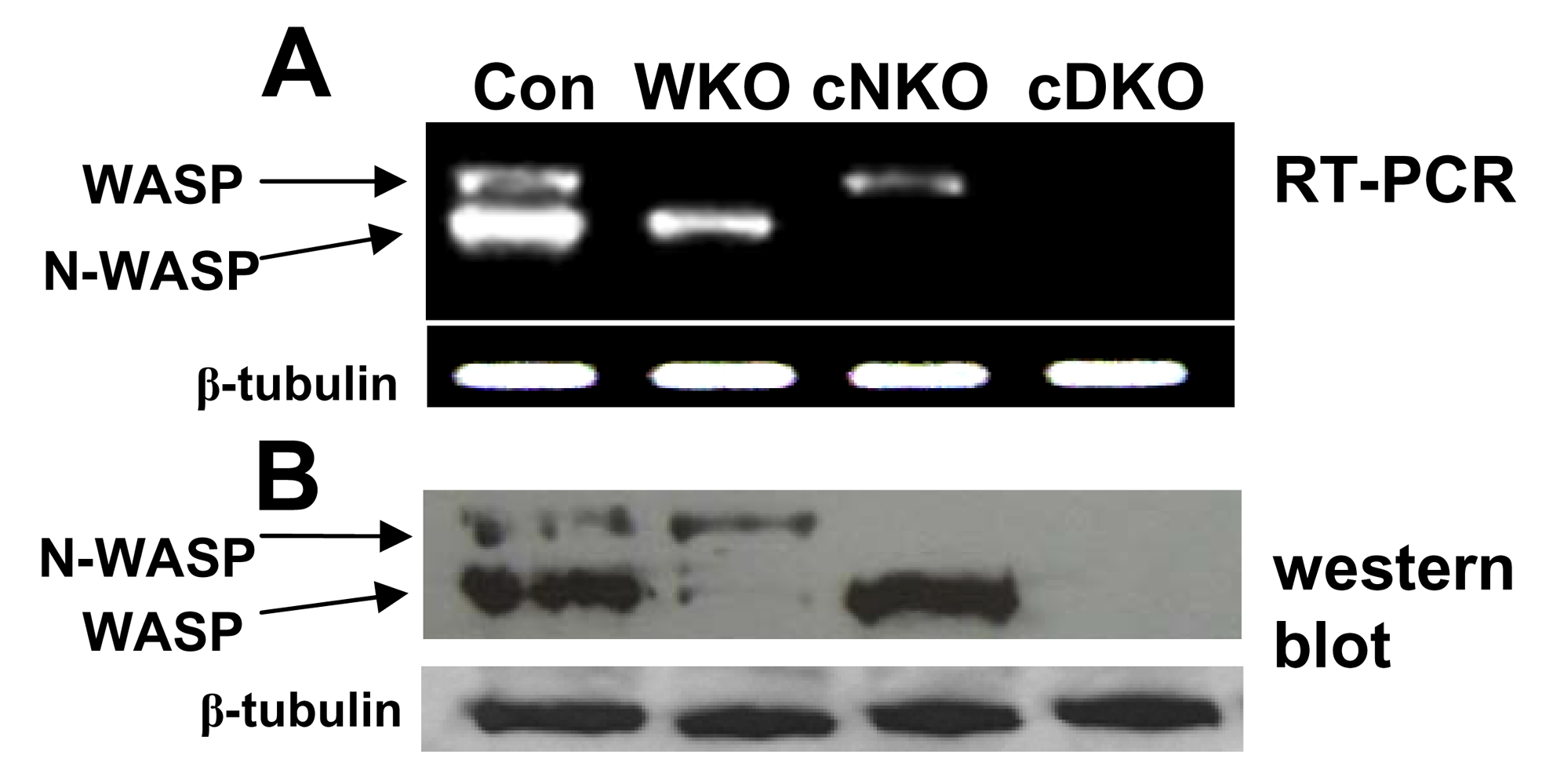

Supplement: Figure S3 — The mRNA and protein expression of WASP and N-WASP in B cells from WKO, cNKO, and cDKO mice. B cells sorted from splenocytes of littermate control, WKO, cNKO, and cDKO mice. (A) The mRNAs were extracted, and RT-PCR was performed. WASP and N-WASP were amplified by PCR using specific primers. (B) Cells were lysed and analyzed by SDS-PAGE and Western blot that were blotted with WASP- and N-WASP–specific antibodies. (TIF) [file pbio.1001704.s003.tif]

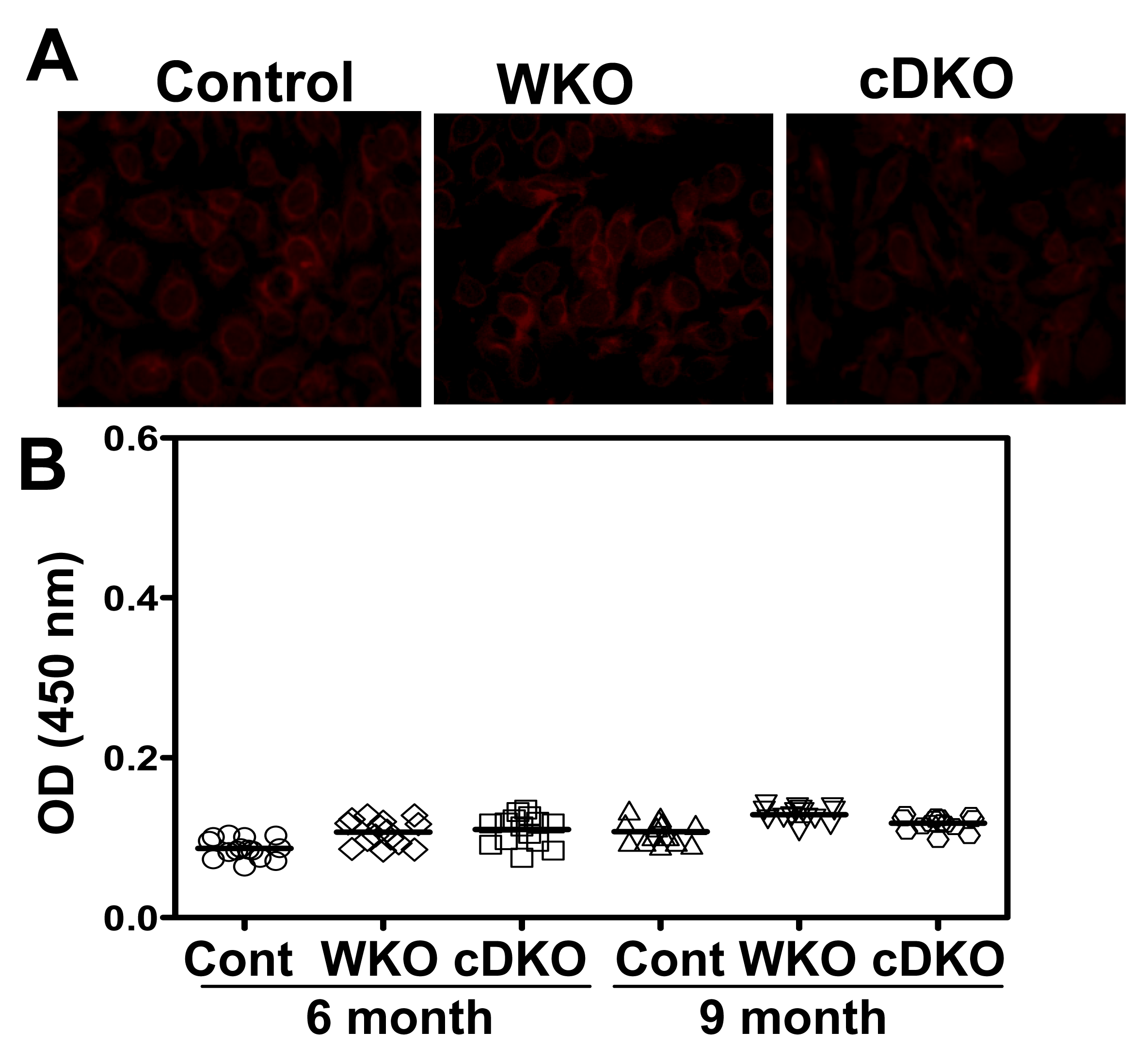

Supplement: Figure S4 — The serum levels of anti-nuclear and anti-dsDNA antibody are not significantly increased in WKO and cDKO mice. (A) Representative images from immunofluorescence microscopic analysis of anti-nuclear antibody in the serum of littermate control, WKO, and cDKO mice of 6 mo old (n = 4). (B) ELISA quantification of anti-dsDNA antibody in the serum of littermate control, WKO, and cDKO mice at 6 and 9 mo old. Each dot represents an individual mouse. n = 15. (TIF) [file pbio.1001704.s004.tif]

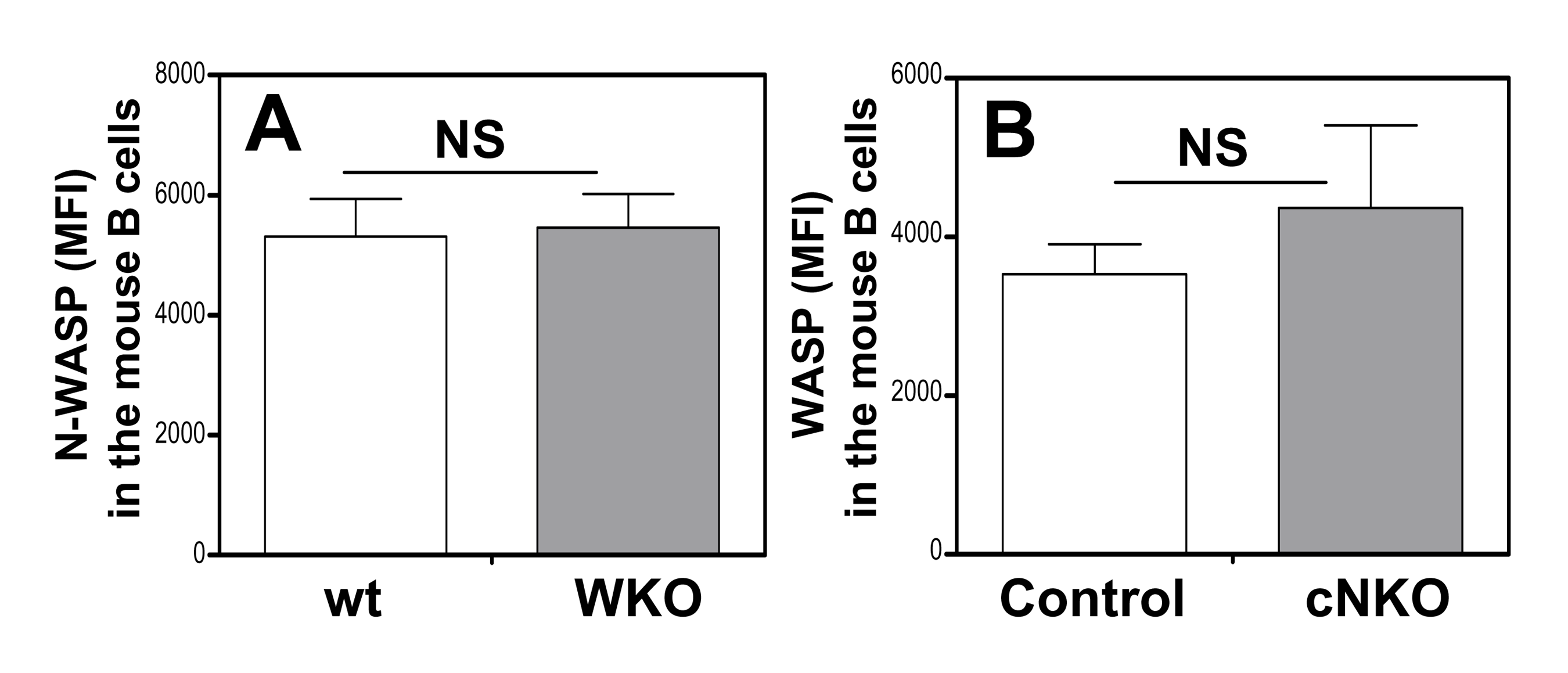

Supplement: Figure S5 — WASP or N-WASP gene knockout does not affect the overall expression levels of N-WASP and WASP. Splenic B cells from wt, littermate control, WKO, and cNKO mice were fixed, permeabilized, stained for WASP and N-WASP, and analyzed using flow cytometry. The average MFI (±SD) was generated from three independent experiments. (TIF) [file pbio.1001704.s005.tif]
